# Calls of the little auk (*Alle alle*) chicks reflect their behavioural contexts

**DOI:** 10.1371/journal.pone.0299033

**Published:** 2024-02-23

**Authors:** Anna N. Osiecka, Elodie F. Briefer, Dorota Kidawa, Feliksa Żurawska, Katarzyna Wojczulanis-Jakubas

**Affiliations:** 1 Department of Vertebrate Ecology and Zoology, Faculty of Biology, University of Gdańsk, Gdańsk, Poland; 2 Behavioural Ecology Group, Section for Ecology and Evolution, Department of Biology, University of Copenhagen, Copenhagen, Denmark; McGill University, CANADA

## Abstract

Animal vocalisations can often inform conspecifics about the behavioural context of production and the underlying affective states, hence revealing whether a situation should be approached or avoided. While this is particularly important for socially complex species, little is known about affective expression in wild colonial animals, and even less to about their young. We studied vocalisations of the little auk (*Alle alle*) chicks in the Hornsund breeding colony, Svalbard. Little auks are highly colonial seabirds, and adults convey complex behavioural contexts through their calls. We recorded chick calls during two contexts of opposite affective valence: handing by a human, and while they interact with their parents inside the nest. Using permuted discriminant function analysis and a series of linear mixed models, we examined the effect of the production context/associated affective valence on the acoustic parameters of those calls. Calls were reliably classified to their context, with over 97% accuracy. Calls uttered during handling had higher mean entropy, fundamental frequency, as well as lower spectral centre of gravity and a less steep spectral slope compared to calls produced during interactions with a parent inside the nest. The individuality of handling calls, assessed by information content, was lower than the individuality of calls uttered in the nest. These findings suggest that seabird chicks can effectively communicate behavioural/affective contexts through calls, conveying socially important messages early in development. Our results are mostly in line with emotional expression patterns observed across taxa, supporting their evolutionary continuity.

## Introduction

Acoustic communication plays a crucial role for many animals, and it can be especially well-developed in socially complex species [1, 2]. Certain information about the environment or behavioural context can be particularly important to communicate to other group members. For example, sharing information about predator presence or food location can be key for colonial or cooperating animals.

Behavioural contexts associated with functionally important stimuli, such as food or threats, can trigger short-term responses associated with physiological, behavioural and cognitive changes, termed ‘emotions’ or ‘affective states’ [3]. Affective states in non-human animals are commonly measured along two fundamental dimensions [4]: arousal (bodily activation) and valence (positive or negative) [5, 6]. They are associated with neuro-physiological, behavioural and cognitive changes [3, 7], serving as guides for adaptive behaviour. In other words, stimuli that promote fitness (e.g. food, caretakers, mating opportunities) are predicted to evoke positive states and typically result in approach behaviour, whereas threatening stimuli (e.g. predators, fights, perilous conditions) are predicted to elicit negative states and generally lead to avoidance [5]. It is important to note that, when discussing emotions one does not necessarily refer to complex feelings, but rather the very basic triggers of behavioural responses [3–7]. Emotional contagion, i.e. transfer of affective states to others, is a key behavioural aspect in socially living animals [8], used to alert the group about both positive and negative contexts, but also maintain social bonds [8–10]. Vocalisations are a powerful means to convey affective ‐ and therefore behavioural ‐ contexts to others [11–13]. Changes in acoustic signals reflecting those contexts can be perceived within [14] and even across species [15], guiding appropriate responses towards the producer of the vocalisation or the situation in which it finds itself [8, 16, 17]. However, dynamic information such as behavioural or emotional contexts may interfere with static information conveyed in calls, such as identity of the caller [18].

The little auk (*Alle alle*) is a highly colonial seabird that maintains long-term social bonds [19]. Little auk pairs produce one egg per year [19], so that no sibling competition occurs. Both partners contribute to and coordinate their parental efforts [20]. Adults of this species have a complex vocal repertoire (eight different call types used during mating and incubation) [13], and convey contextual and emotional information through their calls [13]. Little auk chicks are known to produce one call type during social interactions–the *begging* call [21]–used while they wait for their parent’s return to the nest and during interactions with the parents. The *begging* call is highly individually specific [21] and its acoustic parameters change as the chicks grow [21]. We have also observed some chicks producing calls as they were being handled for ornithological procedures, yet these calls (from here on, the *handling* calls) were not previously described. Aside from this, little is known about the vocal behaviour of little auk chicks [21], or vocal ontogeny in this species.

In this study, we examined whether vocalisations uttered by young little auk chicks already reflect the behavioural contexts of their production ‐ and, if so, which acoustic parameters encode this information. We also investigated whether the information content of chick calls varies between those contexts.

## Methods

### Ethics statement

Fieldwork was performed under permission from the Governor of Svalbard (17/00663-2, 20/00373-8), following Association for the Study of Animal Behaviour guidelines for animal studies [22]. Birds were handled by a licensed ringer (KWJ, permit no. 1095, type: C, issued by Museum Stavanger, Norway).

### Site, subjects, and set-up

All data were collected in the little auk colony in Hornsund, Spitsbergen (77°00′ N, 15°33′ E). Recordings were made over the chick rearing period of the 2017 and 2021 breeding seasons, around the 7^th^ day (approximately 5 to 8 days) of chicks’ life.

In 2017, audio material was collected via an Olympus ME-51S stereo microphones (frequency response 100–15,000 Hz) placed inside 16 nests in such a way as to not disturb the birds’ normal activities [21]. Each microphone was connected to an Olympus LS-3 or LS-P4 digital voice recorder (sampling rate 48 kHz, 16 bits) placed outside of the nest and hidden under a rock to prevent both damage to the equipment and disturbance to the animals. This was paired with video recordings to control for presence/absence of the parent. Only calls produced at times during which a parent was present in the nest were selected for this study.

In 2021, chicks were recorded during handling (weighting) via a hand-help recorder (Olympus LS-12) with a built-in microphone. Chicks were not specifically stimulated to vocalise. Therefore, all recorded calls represent ‘spontaneous’ vocalisations. Note, however, that not all handled chicks vocalised–and among those who did, the call production rate differed greatly (Table 1). Chick sex was unknown in either group–however, note that little auk vocalisations are not affected by the caller’s sex [23].

**Table 1:** Number of calls per individual and production context.

Context	*begging*		*handling*	
	individual	no. calls	individual	no. calls
	N_01	224	H_01	1
	N_02	55	H_02	9
	N_03	293	H_03	4
	N_04	124	H_04	2
	N_05	26	H_05	9
	N_06	143	H_06	1
	N_07	216	H_07	2
	N_08	67	H_08	32
	N_09	81	H_09	11
			H_10	5
			H_11	69
			H_12	13
			H_13	1
			H_14	1
			H_15	7
			H_16	32
			H_17	1
			H_18	3
			H_19	1
			H_20	1
			H_21	1
**Total**	**9 individuals**	**1229 calls**	**21 individuals**	**206 calls**

The effect of the employed equipment (internal vs. external microphone) and recording conditions (inside a rocky burrow vs. in open air) on the acoustic parameters of the recorded calls was tested in an additional experiment (see S1 File). This included broadcasting the calls in conditions mimicking the recording conditions, and re-recording them with both internal and external microphones. Results showed that while the recording set-up had a significant influence over the recorded parameters of the calls, it did not interfere with the overall classification accuracy. See S1 File for a detailed description of this experiment.

**Fig 1:**
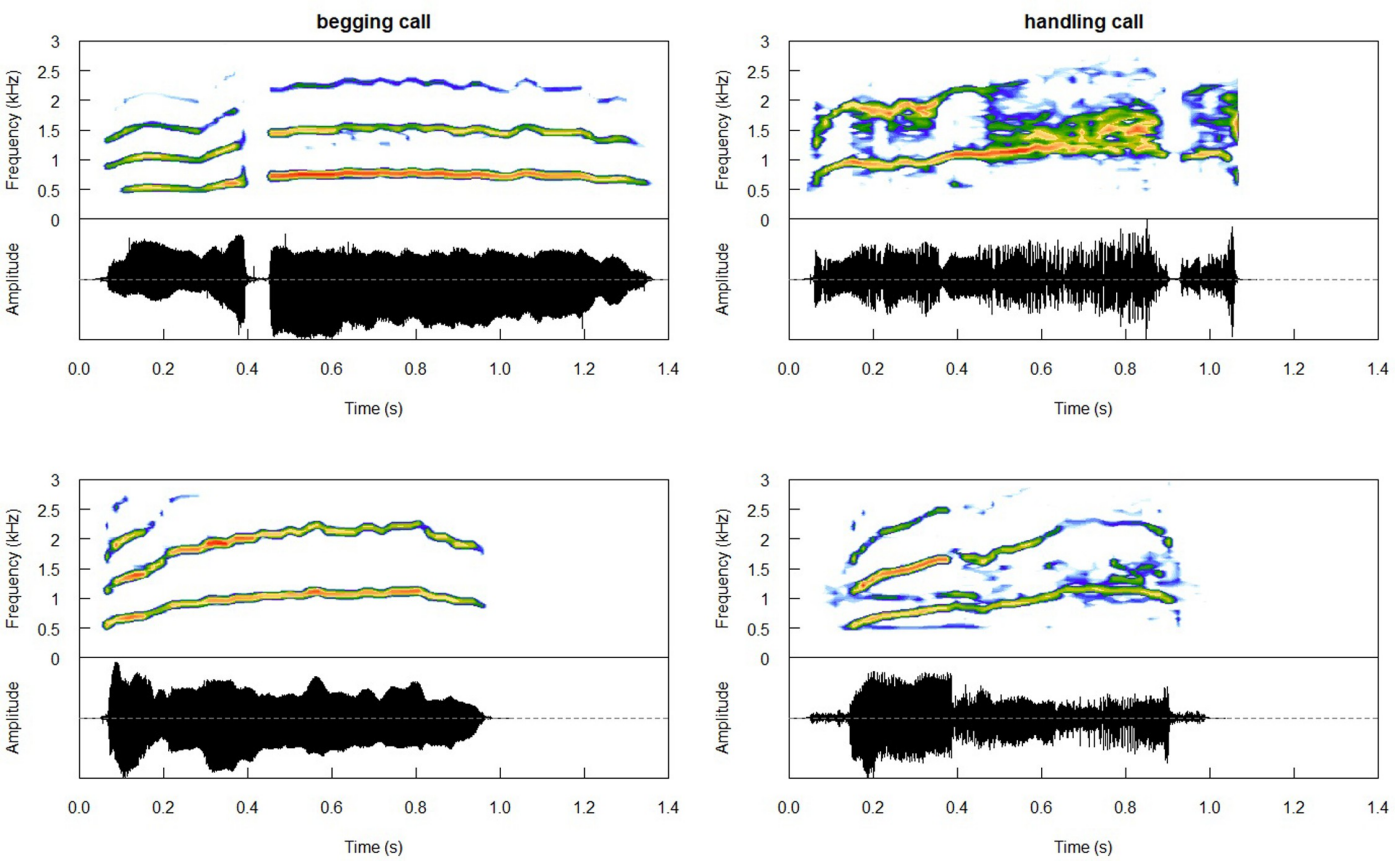
Spectrograms (*seewave* package [36]) of the calls produced inside the nest when interacting with a parent (left panel) and during handling (right panel). Each call was produced by a different chick.

We manually extracted all good quality calls found within the recordings using Raven Pro 1.6.4 (Cornell Lab of Ornithology, Ithaca, USA), assigning them to individual chicks and production contexts (during handling versus inside the nest with a parent; further on *handling* and *begging*, respectively). Both the number of successfully recorded chicks and number of vocalisations produced by those varied greatly between contexts and individuals (Table 1). Since the *begging* and *handling* calls are rather similar to the human ear and are not always obviously different upon a visual inspection of their spectrograms (Fig 1), for the purpose of this study, we decided to treat them as a single call type emitted in two behavioural contexts.

The two behavioural contexts were assigned putative affective valence based on threats/promoters of fitness–i.e., situations that the animals should be motivated to approach or avoid [3, 6, 24]. Therefore, since *handling* likely represents response to a threat, it was assigned putative negative valence (avoidance). There is no sibling competition in the little auk (since broods are composed of one egg only) [19] and adults feed any chick they find in their nest chamber without adverse reaction to it [21]–therefore, *begging* calls were assigned putative positive valence (approach). Note that this approach does not correct for subtler behavioural contexts, such as e.g. frustration related to hunger or prolonged waiting, and aims to refer to the overall valence of the state as positive or negative contexts only. Also note that the use of terms “approach/avoidance” does not necessarily translate into a physical action, but rather an elicited reaction to, or internal motivation to approach/avoid the stimulus.”

### Analysis

All analyses were performed in R environment (v. 4.1.3) [25]. Calls were analysed using the *analyze* function (*soundgen* package [26]) with the following settings: sampling rate = 48000, dynamic range = 60, pitch floor = 800, pitch ceiling = 3500, step = 5. The following parameters were extracted: sound duration, mean entropy, frequency value at the upper limit of the first (Q25%), second (Q50%), and third (Q75%) quartiles of energy, mean fundamental frequency (mean *f*0), and spectral slope. These were selected as standard acoustic parameters used in the studies of vocal expression of affect.

To investigate the difference in the vocal expression between the two contexts/associated putative affective states, we performed a permuted discriminant function analysis (pDFA [27]), pooling all available vocalisations from all individuals and from across the two contexts together (1435 calls in total, Table 1). The use of pDFA allowed us to test the effect of contexts/affective valence (test factor) on the extracted acoustic parameters of the calls (input variables), while controlling for repeated measures of the same individuals (control factor) and the unbalanced dataset [27]. A pDFA with nested design was conducted using the *pDFA*.*nested* function (R. Mundry, based on function *lda* of the *MASS* package [28]). The pDFA used all available subjects (30 individuals) to derive the discriminant function. We ran a total of 1000 permutations for the analysis.

To understand the direction of changes in call parameters, we additionally ran a series of linear mixed models (LMM; *lmer* function, *lme4* package [29]), using each parameter as a response variable (one model per parameter), context/affective valence as a fixed factor, and chick identity as a random factor to control for repeated measures of chicks within contexts (since many chicks produced several calls). Data distribution was tested using Q-Q plots (*qqnorm* function, *stats* package [30] To conform to normal distribution, mean entropy values were log-transformed as: log(*mean entropy*+1-min(*mean entropy*)). The model residuals for all other parameters did not deviate from a normal distribution. To extract the p-values of the LMMs, we used the *PBmodcop* function (*pbkrtest* package [31])), comparing models with and without context included.

To investigate whether calls associated with the two contexts carry a different individual information load, we used individuals who produced at least five calls (i.e., nine individuals per context), and randomly selected five calls for each of them. We calculated the Kaiser-Meyer-Olkin criterion (function *KMO*, *EFAtools* package [32])), which confirmed the data were appropriate for a principal components analysis (PCA; overall KMO score: 0.73). We then ran a PCA including all extracted acoustic parameters (function *prcomp*, *stats* package [30]), and used all 11 principal components to calculate Beecher’s information statistic [33] (Hs; function *calcHS*, *IDmeasurer* package [34], , which automatically provides Hs values for both all and significant variables only). Beecher’s statistic provides an measure of the level of individuality coded within a signal [33] and is a robust, standard method allowing for cross-species comparisons [35]. The Hs values stand for bits of information, and can be translated as the approximate number of individuals that can theoretically be distinguished using a given signal, calculated as 2^Hs.

## Results

**Fig 2:**
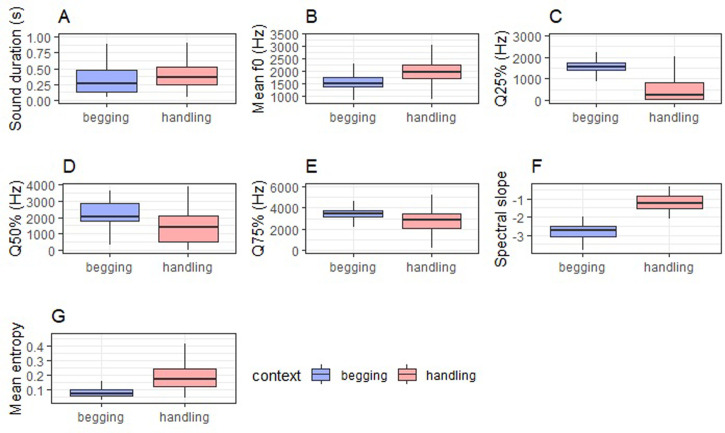
Effect of the behavioural context on the acoustic parameters of the calls. Plots use accessible scientific colour palettes [37–39].

Calls could be reliably classified to their context of production by the pDFA above chance levels (p<0.005; Table 2 and Fig 1). There were significant differences between contexts in the mean entropy, mean *f*0, Q25%, Q50%, and spectral slope, but not in the call duration or Q75% (Table 3 and Fig 2). Spectral slope was steeper in the *begging* calls (Table 3 and Fig 2). Q25% and Q50% were both lower in the *handling* calls, and the mean *f*0 and mean entropy were higher (i.e. less tonal) in the *handling* calls compared to the *begging* calls (Table 3 and Fig 2).

**Table 2:** Results of the permuted discriminant function analysis for the *handling* and *begging* calls, using 7 raw acoustic parameters. Significance level indicated with asterisks.

Result	
No. context categories (levels of test factor)	2
No. individuals	30
Total no. calls.	1435
Correctly classified (%)	99.22
Chance level (%)	78.65
*P* value for classified	0.02*
**Correctly cross-classified (%)**	**97.32**
**Chance level for cross-classified (%)**	**59.64**
Relative cross-classification level	1.63
*P* value for cross-classified	0.001***

**Table 3:** Results of the linear mixed models testing the effect of production context on raw acoustic parameters. Letters (A)-(G) refer to the respective panels in Fig 2.

		*Predictors*		*Scaled residuals*					p-value	Interpretation
		begging	handling (intercept)	Min	1Q	Median	3Q	Max		
**Sound duration (s)**	*Estimates*	**-**0.040	0.347	-4.77	-0.37	-0.04	0.33	8.87	>0.05	No effect (A)
	*Std*. *Error*	0.063	0.036							
	*t-value*	-0.639	9.509							
**Mean *f*0 (Hz)**	*Estimates*	-317.080	1883.050	-4.27	-0.50	0.07	0.60	3.69	<0.05*	Increase during handling (B)
	*Std*. *Error*	135.73	79.930							
	*t-value*	-2.336	23.560							
**Q25% (Hz)**	*Estimates*	913.700	572.000	-3.81	-0.53	-0.06	0.56	4.87	<0.001***	Decrease during handling (C)
	*Std*. *Error*	188.400	110.700							
	*t-value*	4.850	5.166							
**Q50% (Hz)**	*Estimates*	742.200	1518.600	-4.98	-0.42	-0.03	0.40	7.47	<0.01**	Decrease during handling (D)
	*Std*. *Error*	285.700	163.100							
	*t-value*	2.597	9.313							
**Q75% (Hz)**	*Estimates*	-171.5	3535.500	-4.92	-0.40	0.02	0.43	9.31	>0.05	No effect€(E)
	*Std*. *Error*	460.100	262.00							
	*t-value*	-0.373	13.494							
**Spectral slope**	*Estimates*	-1.552	-1.240	-4.38	-0.56	0.05	0.57	5.16	<0.001***	Less steep during handling
	*Std*. *Error*	0.165	0.093							
	*t-value*	-9.405	-13.305							
**Mean entropy**	*Estimates*	-0.128	0.172	-5.42	-0.32	-0.01	0.29	7.74	<0.001***	Increase during handling, i.e. less tonal (G)
	*Std*. *Error*	0.020	0.011							
	*t-value*	-6.587	15.497							

**Fig 3:**
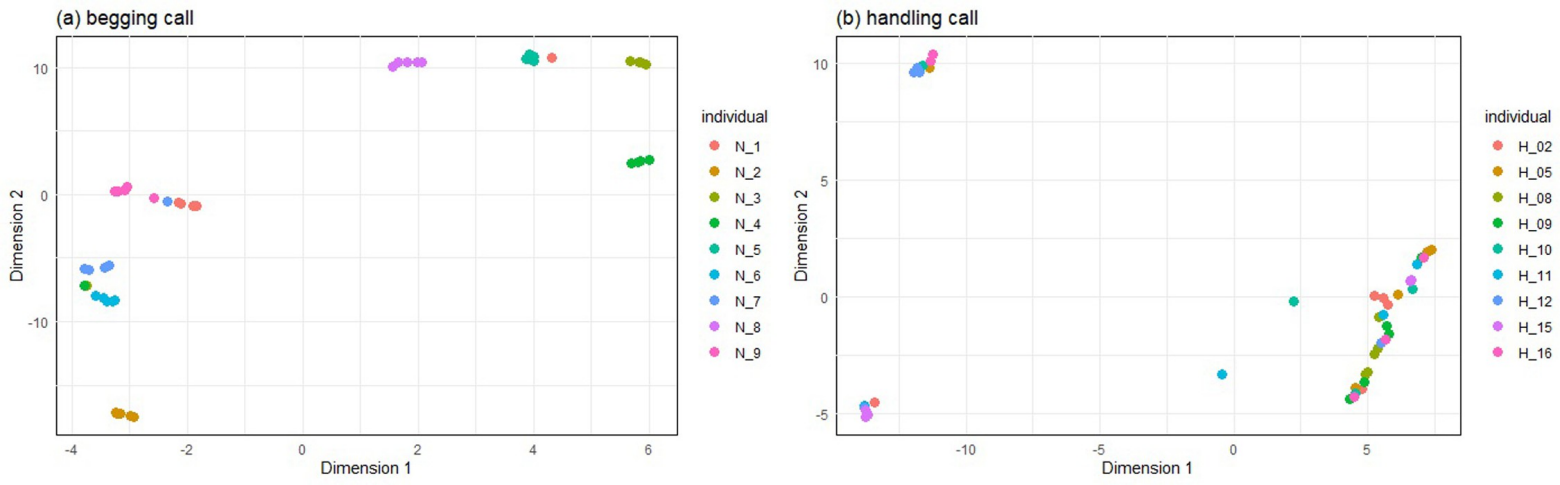
S-UMAP classification to individual for the a) *begging* and b) *handling* calls. While *begging* calls cluster quite well to individuals, this is not the case when the *handling* calls are considered. Plotted using *umap* function of package *uwot* [40], with five nearest neighbours, using all PCA scores of calls emitted by individuals with at least five recorded calls.

Calls uttered during interaction with the parent inside the nest had a Hs = 5.26 (for both all and significant variables), while calls uttered during handling had a Hs = 0.79 for significant variables and Hs = 1.27 when including all variables (Table 4). In other words, calls uttered in during handling were less individually specific than the *begging* calls (Fig 3).

**Table 4:** Beecher’s information statistic of the *begging* and *handling* calls indicates, that call become much less individually specific in a situation of distress.

Context	Hs all	Hs significant	Meaning
*begging*	5.26	5.26	Theoretically allows distinction of at least 38 individuals
*handling*	1.11	0.45	Theoretically allows distinction of at least 1–2 individuals

## Discussion

Communicating behavioural contexts and the underlying affective information is particularly important for socially complex species. We investigated whether calls of seabird chicks carry cues to two behavioural contexts of opposite affective valence. Our results showed that within the first week after hatching, chicks of the colonial seabird, little auk, already have the potential to effectively convey in their calls, information about the context in which they find themselves, which is likely associated with a specific affective valence. Their expression patterns follow the general trends observed across taxa [6, 41–47].

Vocal expression of emotional valence has previously been described in adult little auks [13]. Some of the parameters that encode behavioural or affective information in little auk chick calls align with those observed in adults [13]. Similar to adult vocalisations, chick calls associated with a positive context exhibited a steeper spectral slope and higher spectral centre of gravity compared to those associated with a negative context. However, while adult calls uttered in a positive context (interaction with the partner) show a higher mean fundamental frequency (mean *f*0) and a shorter sound duration [13] compared to negative contexts, in chick calls, we observed a lower mean *f*0 in the positive context and no context effect on sound duration. Those differences can potentially arise from the parameters being related to emotional arousal rather than valence–arousal being a dimension [4] we could not reliably measure, or might be due to differences in emotion expression between call types, as often observed in other species [44, 46]. For example, while a threat presence (handling) and presence of a parent returning from a long foraging trip might well trigger emotions of opposing valence, they could both result in high arousal. At the same time, while begging calls are related to a context that should activate the pleasant-appetitive motivational system and elicit approach, they can also reflect some levels of frustration [48] and stress due to e.g. hunger [49], or prolonged waiting for the parent [50]. While handling by a human can be reliably considered a stressor [51–53], also such prolonged hunger and frustration can increase stress hormone levels [54]–and these, in turn, might reflect on the begging behaviour (e.g. rate of begging calls) [55, 56]. It is therefore possible that the observed differences result from behavioural and emotional complexity not accounted for in this study. Dedicated investigations using measures of arousal (such as heart rate or corticosterone levels) and detailed behavioural observations (e.g. accounting for the time since last feeding) would be helpful to better understand affective expression patterns in the species.

Chick calls associated with a positive context had a reduced mean entropy compared to calls of negative context. This parameter was not examined in adults in our previous study [13]–to allow comparisons, we ran an additional analysis on the previously published data, the results of which showed increased entropy in calls associated with a negative valence across call types (S1 File). High values of entropy reflect less tonal and therefore more chaotic/noisy calls. Therefore, the high entropy values of the calls produced during handling suggest that those calls are more noisy and less tonal than calls produced at the nest. For animals that encode their identity in the spectral parameters of their calls, like the little auk [21, 57], increased noisiness of the signal will likely result in a loss of such information. That is, more chaotic calls may potentially carry less bits of information in their structure. Indeed, distress calls showed a much lower potential for individuality coding than calls associated with a positive context. This could have significant social consequences, such as impaired individual recognition, and suggest that more critically important messages are conveyed instead, e.g. presence of a threat. Some loss of individual information has previously been shown in other species, depending on the valence [58] or arousal [59, 60] of the caller. If distress calls are aimed at the stressor (in this case: the human researcher handling the chick) and intended e.g. to induce release [61], losing the static individual information should not be problematic to the caller [18]. In fact, such loss can be beneficial in situations of distress, if one can catch the attention of any available rescuer, not just their caretaker’s [62]. Surprisingly, cross-fostering experiments have shown that little auk parents will feed any young found in their burrow, which also suggests that individual information in chicks calls might be functionally unimportant in this species [21], or simply overridden by situationally critical information, such as communicating presence of a threat with a noisy signal.

Increased noisiness of acoustic signals is indeed often related to increased arousal or negative valence [11, 41, 42]. In birds, it has been shown to reflect wellbeing of commercially bred chicks [41] (*Gallus gallus domesticus*), where it has been suggested as a useful tool in welfare assessment. Being a parameter that reflects a level of disorder rather than a specific value (such as e.g. mean *f*0) that may change as an animal grows or ages, it can prove a reliable indicator of an individual’s emotional state without prior knowledge of its age or weight. As such, spectral entropy is a promising parameter for social communication in large groups.

Little auk calls are highly individually specific [21, 57], but also reflect the size and overall body condition of the calling chick [21] (similarly to those of the Wilson’s storm petrel, *Oceanites oceanicus* [63]). Here, we did not correct for the chicks’ size, yet the recordings used come from chicks of roughly the same age (5–8 days after hatching), which corresponds to comparable body sizes [21]. While we have employed methods that correct for multiple testing of the same individuals, we acknowledge that this study would have benefited from testing the same individuals in both behavioural contexts. However, this study aimed to maximise the use of already existing data to limit the disturbance caused by research activities and avoid unnecessary handling [64]. Here, we took advantage of the available recordings made inside the nests in 2017 [21], supplementing them with additional recordings taken during chick handling for population monitoring in 2021. Little auk handling calls are produced in a very unpredictable manner, and few animals vocalise when handled [13]–this is true for both chicks and adults. Without external stimulation by the researcher, which we avoid, collecting recordings from both contexts for the same individuals usually proves unsuccessful, and it cannot be guaranteed that an animal recorded in one context will vocalise in another. As a result, the available recordings presented in this study are the only chick vocalisations available to investigate context-related changes in call structure. Note that we did not account for the chicks’ sex in our analyses, since this information was not available. However, since adult little auks show no sex differences in their calls [23], we are confident that this factor does not significantly impact our results.

An important caveat of this study is that we only had access to two behavioural contexts (one context per putative affective valence) with their associated calls–which may represent either a gradation of one call type, or two distinct calls types. This is because the vocal activity of the young chicks in this species is rather limited in contexts–only the *begging* calls have been previously observed in the young chicks during the nesting period [21]. The data presented here enrich our knowledge of the chicks’ behaviour through adding the negative contexts. They are also all that was possible to record of the young chicks’ vocalisations within the logistical and ethical limitations, and as such present an important contribution. We also were not able to compare the stability of the two calls across the season (within individuals) and years (across individuals). The little auk *begging* calls change as the chicks grow [21], and it would be ideal to follow the affective expression over ontogeny–unfortunately, such data are not currently available. On the other hand, seabird calls tend to be individually stable over the years [57, 65, 66], and we do not expect repertoire changes to occur within small timescales. The *begging* and *handling* calls are not obviously different upon a visual inspection of their spectrograms (Fig 1). Adult little auks use some of their calls across multiple contexts [13], and this is likely true also in case of the chicks. Nevertheless, our results may in fact reflect call type differences. In either case, the vocalisations used in this study reflect two very different behavioural contexts clearly conveyed by the sender, and we are confident that they can be safely interpreted as such. Note that by discussing conveying the behavioural or affective context, only the signal structure is meant: this study cannot assume the intended receiver of this signal (i.e., whether a vocalisation is directed at other auks or the threat itself [60]) or whether and how this intended receiver interprets it. Importantly, the expression patterns observed in this study align with both the patterns observed in the adults of the species [13], and the general trends seen in other groups taxa [6, 41–47].

Inquiries into emotions of non-human animals requires taking a perspective relevant to the animal, and using robust, conservative measures [67]–ideally, physiological measures should be used, but external measures such as vocal expression patterns prove extremely helpful when such measures are impossible to take [11, 12]. While affective states in birds have been studied in depth in some species under controlled, experimental conditions [43, 48, 68], data on non-captive animals are still scarce. Our results contribute to the growing body of research on emotions in the wild, and mostly support the general trends seen in affective signals across the vertebrate evolutionary lines [6, 41–47, 62].

## Conclusions

Calls of the little auk chicks carry information about their production contexts early in ontogeny, i.e. within the first week after hatching. Acoustic differences between calls uttered during handling (negative context/elicited avoidance) and inside the nest during interaction with a parent (positive context/elicited approach) are mostly in line with emotional expression patterns observed across taxa, supporting its evolutionary continuity. Our findings present the first evidence of affective expression in seabird chicks, and suggest that little auk chicks might effectively convey socially important messages early in development.

## Supporting information

S1 File Full descriptions of the two additional analyses performed for this study. (DOCX)

## List of abbreviations

f0: fundamental frequency
LMM: linear mixed model
mean *f0*: mean fundamental frequency
pDFA: permuted discriminant function analysis
Q25%: frequency value at the upper limit of the first quartile of energy
Q50%: frequency value at the upper limit of the second quartile of energy
Q75%: frequency value at the upper limit of the third quartile of energy
