# Supplementary material for: Calls of the little auk (Alle alle) chicks reflect their behavioural contexts
Source: PLoS One. 2024 Feb 23;19(2):e0299033. doi: 10.1371/journal.pone.0299033 (PMC10889865; doi:10.1371/journal.pone.0299033)
Supplement: S1 File — (DOCX) [file pone.0299033.s001.docx]

**Supplementary materials**

1. **Testing for the effect of the equipment and recording conditions**

To rule out that the recording equipment employed in the two field seasons and the recording conditions (inside a rocky burrow vs. in open air) had an influence the acoustic parameters of the recorded calls, and could hence explain the differences between the two contexts used in this study, we performed an additional experiment.

For each contexts, we selected one vocalisation (semi-random selection of a good quality signal) per individual (n = 9 birds) per context (vocalising inside the nest with a parent vs. vocalising during handling). These 18 vocalisations were then played back and recorded with the same equipment as used in the field, in the four following settings:

1. Recording with an external microphone, in open air
2. Recording with an external microphone, in a wooden box
3. Recording with a built-in microphone, in open air
4. Recording with a built-in microphone, in a wooden box

Each vocalisation was played back three times in each setting. The acoustic properties of the re-recorded signals were extracted using the *analyze* function of the *soundgen* package[26] (dynamic range = 60, pitch floor = 800, pitch ceiling = 3500, step = 5[13]). The following parameters were extracted (i.e. the same parameters as in the rest of the study): sound duration, mean entropy, frequency value at the upper limit of the first (Q25%), second (Q50%), and third (Q75%) quartiles of energy, mean fundamental frequency (mean *f*0), and spectral slope.

We performed two permuted discriminant function analyses (pDFA[27]; R. Mundry, based on function *lda* of the *MASS* package[28]), pooling the vocalisations from the two contexts together, using the vocalisation identification number as a control factor, and (1) recording set-up (i.e. open/closed environment and with/without external microphone) as the test factor; incomplete pDFA; and (2) production context as the test factor; nested pDFA. We ran a total of 1000 permutations per analysis.

The following results were obtained:

1. recording set-up as the test factor: p-value for selected: 0.001; p-value for cross-classified: 0.001; expected percent correctly classified: 47.01; expected percent correctly cross-classified: 67.60; percent correctly classified: 80.60; percent correctly cross-classified: 67.60.
2. production context as the test factor: p-value for selected: 0.001; p-value for cross-classified: 0.004; expected percent correctly classified: 72.62; expected percent correctly cross-classified: 62.05; percent correctly classified: 95.15; percent correctly cross-classified: 87.49.

Resulting accuracy shows that while the recording set-up did have a significant influence over the recorded parameters of the calls, this influence was lower than that of the production context, and vocalisations could be reliably classified to their production context independently of the equipment and recording environment.

The raw data generated in this experiment are available at: <https://osf.io/nwpfk/?view_only=a1d5577723424699b12b1c707a5369cc>

1. **Entropy in negative calls of adult little auks**

To allow comparisons among groups – and include more call types – we performed an additional analysis of adult calls with assigned positive and negative valence, using recordings from Osiecka *et al.* 2023[13].

The analyses was performed in R environment (v. 4.1.3)[25]. Calls were analysed using the *analyze* function (*soundgen* package[26]) with the following settings: sampling rate = 48000, dynamic range = 60, pitch floor = 500, pitch ceiling = 2000, step = 5[13], extracting mean entropy values.

Calls were assigned positive and negative valence based on the call type/production context[13]. We ran a linear mixed model (LMM; *lmer* function, *lme4* package[29]), using mean entropy as a response variable (one model per parameter), putative valence as a fixed factor, and call type as a random factor to control for repeated measures of the same call types. Data distribution was tested using Q-Q plots (*qqnorm* function, *stats* package[30]. To extract the p-value, we used the *PBmodcop* function (*pbkrtest* package[31]), comparing models with and without valence included.

The putative affective valence had a significant effect on the mean entropy of the calls (p<0.001***; Supplementary table 1, Supplementary Figure 1). Calls assigned negative valence showed higher entropy compared to calls assigned positive valence (Supplementary Figure 1).


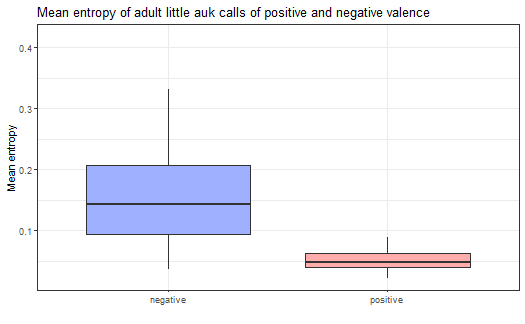


**Supplementary Figure 1.** Effect of the affective valence on the mean entropy of adult calls. *Positive* calls include *clucks*, and *negative* calls include *handling* and *terror* calls[13]. Plots use accessible scientific colour palettes[37–39].

**Supplementary Table 1.** Results of a linear mixed model investigating the effect of valence on the mean entropy of adult little auks’ calls.

|  |  | *Predictors* | | *Scaled residuals* | | | | | **p-value** | **Interpretation** |
| --- | --- | --- | --- | --- | --- | --- | --- | --- | --- | --- |
|  |  | **negative (intercept)** | **positive** | **Min** | **1Q** | **Median** | **3Q** | **Max** |  |  |
| **Mean entropy** | *Estimates* | 0.158 | **-**0.099 | -1.64 | -0.60 | -0.16 | 0.32 | 3.51 | <0.001*** | Higher entropy in negative calls |
|  | *Std. Error* | 0.010 | 0.017 |  |  |  |  |  |  |  |
|  | *t-value* | 16.445 | -5.934 |  |  |  |  |  |  |  |
